# What metabolomics has taught us about tomato fruit ripening and quality

**DOI:** 10.1093/jxb/eraf209

**Published:** 2025-05-21

**Authors:** Esra Karakas, Alisdair R Fernie

**Affiliations:** Max Planck Institute of Molecular Plant Physiology, Am Muhlenberg 1, D-14476 Potsdam-Golm, Germany; Max Planck Institute of Molecular Plant Physiology, Am Muhlenberg 1, D-14476 Potsdam-Golm, Germany; INRAE-Bordeaux, France

**Keywords:** GWAS, metabolomics, QTL, tomato development, tomato metabolism, tomato ripening

## Abstract

Tomato (*Solanum lycopersicum*) is a major crop and model species for investigating fruit metabolism, which plays a crucial role in determining flavor, nutritional quality, and ripening. Metabolomics has profoundly expanded our comprehension of tomato fruit ripening and quality by unraveling the intricate biochemical dynamics underlying these processes. Leveraging high-throughput metabolite profiling, researchers have identified critical metabolic pathways governing ripening, encompassing phytohormones, primary metabolism, and specialized metabolism. Integrated metabolomics, in combination with whole-genome sequencing, genome-wide association studies, and quantitative trait locus analysis, provides a comprehensive approach to identifying key genetic and metabolomic determinants of agronomic traits. Here we provide both established and emerging insights into the metabolic networks governing tomato ripening, emphasizing the potential of metabolomics-assisted breeding to enhance fruit taste and nutrition.

## Teaser

This study highlights how metabolomics unveils key metabolic pathways in tomato ripening, offering insights for breeding strategies aimed at improving flavor, nutrition, and overall fruit quality.

## Introduction

Having been established a quarter of a century ago (Oliver *et al.*, 1998), metabolomics can no longer be regarded as being in its infancy but is also not a mature discipline (Kell and Oliver, 2016; Alseekh and Fernie, 2018). Whilst it is used a lot in medical research where it has aided in a number of important advances (Giera *et al.*, 2022), many of the key early advances in metabolomics occurred in microbial and plant sciences (Aharoni *et al.*, 2023). Given that both technical and computation aspects of plant metabolomics have been extensively reviewed elsewhere (Weckwerth, 2003; Obata and Fernie, 2012; Alseekh *et al.*, 2021; Perez de Souza *et al.*, 2021, 2022; Shen *et al.*, 2023; Perez de Souza and Fernie, 2024; Westhoff and Weber, 2024), we will only cover them briefly here. Despite the fact that some groups utilize NMR as a tool for metabolomics (Kruger *et al.*, 2008; Marchev *et al.*, 2021), given the relatively low sensitivity and, as such, the relatively low coverage of the metabolome of this technique, most contemporary metabolomics approaches are carried out by the use of GC or LC coupled to MS. GC-MS offers the advantage of being highly robust and reproducible, with data being highly comparable even if obtained with different machines (Schauer *et al.*, 2005a). It is, however, compromised by its relatively low mass range, being incapable of measuring larger secondary metabolites (Fernie *et al.*, 2004). LC-MS-based metabolomic approaches have become powerful tools in tomato research, enabling the detection of a broad spectrum of tomato metabolomics, including primary and secondary metabolites. Previous LC-MS studies have facilitated the identification of specific metabolites in tomato such as the phenolic profile (Barros *et al.*, 2012), glycoalkaloids (Iijima *et al.*, 2013), and carotenoids (Fattore *et al.*, 2016). Moreover, LC-MS profiling has also revealed that antioxidant activities of various tomato varieties are correlated with their flavonoid content (Otify *et al.*, 2023). LC-MS has a very broad range of mass detection; however, it suffers from considerably higher levels of variance, rendering comparison of data between machines rather challenging (Perez de Souza *et al.*, 2021). Given this, cross-laboratory comparisons of primary metabolite data obtained via LC-MS are easier than of lipophilic compounds or specialized metabolites (for details, see Perez de Souza and Fernie, 2024). Nevertheless, there are considerable recent computational advances that render this less problematic, of particular note being the MassBank suite of programs (Perez de Souza and Fernie, 2024). Indeed, computational resources for metabolomics are vast (Perez de Souza and Fernie, 2024); whilst these largely began as either collections of statistical tools for data processing or databases to support annotation, recent years have seen the development of a wide range of approaches that directly support peak annotation (for reviews, see Perez de Souza *et al.*, 2017, 2022; Mansoor *et al.*, 2024; Mildau *et al.*, 2024). In the remainder of this review, we will discuss how metabolomics has been used as a component of systems biology-based approaches to understand the metabolic shifts that underpin tomato fruit ripening and development. This will be followed by an in-depth discussion of how metabolomics has advanced our understanding of the genetic architecture controlling the accumulation of agronomically important metabolites that influence key fruit traits such as taste and nutritional value. The final section revisits the concept of metabolomics-assisted breeding and its potential to enhance crop quality.

## Defining the metabolic shifts during tomato development and ripening

Given the complex dynamics in the levels of phytohormones, primary and secondary metabolites, color, and texture displayed by tomato fruits as they ripen alongside their transition from being photosynthetic to heterotrophic, it is unsurprising that this developmental pattern has attracted so much attention at the metabolomics level. This process has been extensively reviewed (Carrari and Fernie, 2006; Fraser *et al.*, 2009; Klee and Giovannoni, 2011; Ruan *et al.*, 2012; Tohge *et al.*, 2014; Martínez-Rivas and Fernie, 2023), so we will only highlight a few papers, focusing on those that have not been covered so extensively in previous reviews. Nevertheless, some of the earliest plant metabolomics experiments looked into the metabolic shifts underlying tomato development and ripening (Roessner-Tunali *et al.*, 2003; Fraser *et al.*, 2007). In the first of these, the influence of overexpressing hexokinase on the metabolic shifts occurring during tomato development was examined, revealing that this decreased over developmental time (Roessner-Tunali *et al.*, 2003). More detailed characterizations were found in later studies which also incorporated gene expression analyses (Rohrmann *et al.*, 2011), with the most detailed of these to date being the combined transcriptomic–metabolomic atlas spanning 20 spatio-temporarily distinct tissues across the entire cv. Micro-Tom life cycle (Li *et al.*, 2020). These studies essentially provided a clear catalog of metabolic changes occurring over developmental time and which have been extensively detailed in reviews over the last 20 years (Fraser *et al.*, 2009). However, whilst this type of study provided broad-brush overviews of the changes that occur during ripening and gave hints towards the underlying molecular mechanisms, much greater insight has been achieved by studying mutants and transgenics which are compromised in the expression of one or other of the key proteins involved in the process. In this vein, research has been dominated by studies of the classical tomato ripening mutants *rin*, *nor*, and *NEVER RIPE* (*NR*), as well as the epigenetic mutant *COLORLESS NON-RIPENING* (*cnr*) (Osorio *et al.*, 2011; Beisken *et al.*, 2014). However, the roles of an increasing number of regulatory transcription factors have recently been brought to light by metabolomics, including those for APETALA2a, TGA2.2, ERFs, LATERAL ORGAN BOUNDARIES, and WD40s, and also MADS box proteins (Karlova *et al.*, 2011; Huang *et al.*, 2021; Shi *et al.*, 2021; Deng *et al.*, 2022; Lemaire-Chamley *et al.*, 2022; Zhu *et al.*, 2022). Similarly, the level of hormonal control underlying fruit development and ripening has been revealed to be considerably more complex than previously thought, with roles for abscisic acid (ABA; Bastías *et al.*, 2014; McQuinn *et al.*, 2020; Dominguez *et al.*, 2021), jasmonic acid (Fenn and Giovannoni, 2021), and brassinosteroids (Fenn and Giovannoni, 2021) joining those of ethylene which was previously viewed as the ripening hormone.

In addition to the systems-biology or multi-omics-based approaches, a handful of targeted approaches have provided considerable mechanistic insights into tomato ripening both at the level of individual enzymes and metabolites (Uluisik *et al.*, 2016) and in terms of transcriptional cues (Karlova *et al.*, 2011; Huang *et al.*, 2021; Shi *et al.*, 2021; Deng *et al.*, 2022; Lemaire-Chamley *et al.*, 2022; Zhu *et al.*, 2022). Prior to the advent of metabolomics, tomato fruit ripening was already characterized to involve shifts from starch to soluble sugars (Klann *et al.*, 1993), hormonal fluctuations (Grierson and Tucker, 1983), changes in pigmentation (Went *et al.*, 1942), and ultimately cell wall softening (Grierson and Tucker, 1983). The study of Carrari *et al.* (2006) aligned the expression of key genes that had been demonstrated to contribute to these changes with changes in the levels of a range of primary metabolites and cell wall precursors, identifying organic acids as metabolites with a potentially high degree of control over these changes. In a subsequent set of experiments, researchers manipulated malate levels by inhibiting the expression of either mitochondrial malate dehydrogenase or fumarase. These alterations led to significant changes in tomato fruit biology. Specifically, increased malate levels were associated with lower levels of transitory starch and reduced soluble sugar content. As a consequence, fruits with elevated malate showed lower susceptibility to bacteria at harvest; in contrast, decreased levels resulted in the opposite changes (Centeno *et al.*, 2011). A subsequent study that showed an increase in malate levels following a different genetic intervention resulted in similar phenotypes, thus reinforcing the importance of this metabolite in ripening regulation (Osorio *et al.*, 2013). Similar, studies have revealed roles for the non-proteogenic amino acid GABA (γ-aminobutyric acid) in both tolerance to alkaline saline stress (Wang *et al.*, 2024) and resistance to fruit flies (Li *et al.*, 2023).

A final area that is worthy of further experiments are recent studies evaluating the epigenetic control of fruit ripening (Giovannoni *et al.*, 2017; Zuo *et al.*, 2020). Given that epigenetics both regulates metabolic processes and, under certain conditions, is in itself mediated by metabolites, closer study of this interaction during the course of ripening is surely merited. Taken together, these studies collectively highlight the fact that profiling of tomato fruits at a range of levels has underlined not only already known aspects of their biology but also novel links in the complex networks that govern its functioning. Whilst many of these links remain mere correlations to date—as can be seen by a number of examples described above—several of them have already yielded mechanistic insights and it can be anticipated that many more such insights will be uncovered in the coming years.

## Genetic architecture of tomato fruit metabolism

### Metabolic quantitative trait loci

Most quality traits exhibit continuous variation and are highly influenced by environmental conditions. The genetic variation in these traits is attributed to the combined effects of multiple genes, known as quantitative trait loci (QTLs), which can be mapped onto the genome using genetic markers. A QTL-based approach has been employed to identify genomic regions that control quality traits in processing tomatoes. QTLs associated with fruit weight, soluble solids content, pH, fruit color, and firmness have been identified in several segregating populations from interspecific crosses (Paterson *et al.*, 1988; Goldman *et al.*, 1995; Eshed and Zamir, 1996; Tanksley *et al.*, 1996; Bernacchi *et al.*, 1998; Chen *et al.*, 1999).

The genetic basis of many agronomically important traits has been explored using segregating populations, as previously mentioned. With the availability of the genome sequence, numerous new candidate genes have been identified. However, only a limited number of mutations linked to significant phenotypes or QTLs have been identified and functionally validated to date. Two QTLs associated with fruit weight have been cloned: one corresponds to a cytochrome P450 gene [*fruit weight* (*fw*3.2); Chakrabarti *et al.*, 2013] and the other to a novel protein, the cell size regulator (*fw*11.2) which primarily increases fruit weight by enlarging the pericarp regions (Bardol *et al.*, 2013; Mu *et al.*, 2017). Among the four genes responsible for diversity in fruit shape (Rodríguez *et al.*, 2011), Soyk *et al.* (2017) demonstrated that the fascinated (*fas*) locus, which contributed to an increase in locule number (*lc*) and fruit size during tomato domestication, is due to a regulatory mutation in the CLAVATA3 (*SlCLV3*) gene. This gene interacts with WUSCHEL (SlWUS) in a genomic region containing two single nucleotide polymorphisms (SNPs) associated with the *lc* mutation (Muños *et al.*, 2011). The product of the *OVATE* gene functions as a growth inhibitor and is a member of the Ovate Family Proteins (OFPs) which plays a key role in determining tomato fruit shape. A single nucleotide mutation (G→T) introduces a premature stop codon, altering the shape of the fruit from round to pear shaped (Liu *et al.*, 2002). Wu *et al.* (2015) developed near-isogenic lines (NILs) for the *OVATE* gene and demonstrated that it influences ovary elongation before flowering, leading to elongation at the proximal end of the fruit. Moreover, OVATE affects seed shape, producing seeds that are shorter and wider than those of the wild type (LA1589), indicating a reduction in seed size.

Over the past few decades, both determinate and indeterminate tomato cultivars have been utilized to generate fast-neutron and ethylmethane sulfonate (EMS) mutant collections (Meissner *et al.*, 1997; Menda *et al.*, 2004; Minoia *et al.*, 2010; Okabe *et al.*, 2011; Garcia *et al.*, 2016). Mutant collections from the determinate processing tomato cultivar M82 and the model miniature tomato cv. Micro-Tom, which is well suited for laboratory use, have been systemically screened for numerous phenotypic traits, including yield, plant architecture, leaf shape, and complexity, as well as flower and fruit morphology, color, and ripening (Menda *et al.*, 2004; Saito *et al.*, 2011; Garcia *et al.*, 2016).

Introgression lines (ILs), particularly those derived from crosses between *Solanum pennellii* and *Solanum lycopersicum* (Eshed and Zamir, 1995), have been widely used to map numerous QTLs associated with fruit composition (Schauer *et al.*, 2006, 2008; Alseekh *et al.*, 2015). A unique QTL was found to enhance the harvest index, earliness, and metabolite content (sugars and amino acids) at the heterozygous level in processing tomatoes (Gur *et al.*, 2010, 2011; Toubiana *et al.*, 2012).

Fine mapping experiments have enabled precise localization of QTLs within specific chromosome regions and confirmed the presence of multiple QTLs linked within the same region (Paterson *et al.*, 1990; Frary *et al.*, 2004; Lecomte *et al.*, 2004). For instance, by narrowing the size of introgressed fragments from *S. pennellii*, Eshed and Zamir (1995) identified three linked QTLs controlling fruit weight on a single chromosome arm. Fine mapping is also a critical step in QTL cloning, as demonstrated by the successful cloning of QTL-associated fruit weight (Alpert and Tanksley, 1996; Frary *et al.*, 2000), fruit shape (Tanksley, 2004), and soluble solid content (Fridman *et al.*, 2000, 2004). Another fine mapping approach utilized the *S. pennellii* and *S. neorickii* backcrossed inbred line (BIL) populations to validate the metabolic candidate genes phenylalanine ammonia-lyase and cystathionine gamma-lyase (Brog *et al.*, 2019). ILs were also utilized to fine-map and positionally clone several genes and QTLs of interest. An acid invertase gene (*TIVI*) from *S. chmielewskii* can enhance fruit sugar composition (Klann *et al.*, 1993), while a single amino acid change in the product of the *S. pennellii* allele for Lycopersicum invertase 5 (*LIN5*) produces a kinetically superior enzyme, increasing the net transport of photoassimilate into the fruit (Fridman *et al.*, 2000, 2004).

The power of QTL mapping was further improved by establishing a sub-IL set with smaller introgressed fragments (Gur *et al.*, 2010; Alseekh *et al.*, 2013), However, the initial lines successfully identified a myriad of QTLs associated with fruit traits (Causse *et al.*, 2004), antioxidants (Rousseaux *et al.*, 2005), vitamin C (Stevens *et al.*, 2007), enzyme activities (Steinhauser *et al.*, 2011), and volatile aromas (Tadmor *et al.*, 2002). Moreover, building on the initial population, Ofner *et al.* (2016) established a BIL population that has been maintained through 11 generations of selfing and includes smaller introgressions. The 446 BILs were thoroughly genotyped using the 10k Solcap SNP array. The researchers identified 1049 unique bins across the entire genome, with about half containing <10 genes. By utilizing two well-known QTLs and genes, *fw*2.2 and beta-carotene (B), they showcased the high-resolution mapping capabilities of this population. Additionally, the genome of the *S. pennellii* accession was sequenced (Bolger *et al.*, 2014), and the BILs facilitated the characterization of QTLs related to yield (Soyk *et al.*, 2017), leaf shape (Fulop *et al.*, 2016), leaf thickness (Coneva *et al.*, 2017), and the day-length response (Müller *et al.*, 2016; Soyk *et al.*, 2017). The newly identified *canal-1* tomato mutant, characterized by a variegated leaf phenotype, has been found to influence yield canalization (Fisher and Zamir, 2021). Wijesingha Ahchige *et al*. (2024) indicated that the *canal-1* tomato mutant, linked to the SNOWY COTYLEDON 2 (AtSCO2) protein ortholog, disrupts yield canalization by impairing photosystem assembly, with green leaves compensating via gene up-regulation, while white leaves remain undeveloped, acting as stress-induced carbon sinks (Wijesingha Ahchige *et al.*, 2024). One of the largest novel *S. pennellii* BIL populations (Schmidt *et al.*, 2017) was recently used in epistasis studies which enabled the identification of 80 cases of epistasis for yield-associated traits (Torgeman and Zamir, 2023). The findings on the high mapping resolution of the BILs and the capability to analyze genome-wide epistasis highlight the potential value of this new resource for the tomato research community.

Various studies have linked tomato fruit composition with its physical characteristics and sensory qualities (Baldwin *et al.*, 2008). Flavor is primarily driven by sugar and acid levels, with an optimal balance between these two components being key to taste. Although >400 aroma volatiles have been identified in tomato fruit, only some play a significant role in shaping tomato aroma. Factors such as tomato variety, ripeness, and storage conditions can influence the levels of these volatiles. Tomato fruits contain not only volatiles but a variety of other taste-related compounds, including sugars, organic acids, and amino acids. Nevertheless, breeding tomatoes with enhanced nutrition and robust flavor remains a significant challenge (Tieman *et al.*, 2012; Klee and Tieman, 2013; Zhao *et al.*, 2016; Garbowicz *et al.*, 2018). However, a major QTL regulating malate content was cloned and identified as the Aluminium Malate Transporter 9 (*Sl-ALMT9*) (Ye *et al.*, 2017). A subsequent study revealed that this QTL is also likely to be involved in regulating citrate content in tomato fruits (Zhao *et al.*, 2019). While only a limited number of QTLs associated with sugars and organic acids have been functionally validated, this knowledge provides critical insights into regulatory mechanisms. Additionally, several genes contributing to the variation in volatile compound production have been characterized (Tieman *et al.*, 2006; Klee, 2010; Tikunov *et al.*, 2013). For example, Tieman *et al.* (2006) described a biosynthetic pathway in tomato fruits for 2-phenylethanol and other volatiles derived from phenylalanine, characterizing a small family of decarboxylases (LeAADC1A, LeAADC1B, and LeAADC2) that catalyze the initial step of the pathway. Phenylpropanoid-derived volatiles were subsequently demonstrated to be key contributors to the ‘smoky’ aroma in tomato fruits. The *NON-SMOKY GLYCOSYLTRANSFERASE1* (*NSGT1*) gene, identified through a combinatorial omics approach, is up-regulated during fruit ripening. Reverse genetics confirmed *NSGT1*-mediated glycosylation as the molecular mechanism for the smoky aroma trait (Tikunov *et al*., 2013). Beyond these specific examples, a high number of other candidate genes have been identified to be involved in tomato fruit flavor, including *1-deoxy-D-xylulose 5-phosphate synthase*, *QR*, *LOXA*, *loxc*, *loxf*, *ADH1*, *ADH2*, *SlSAMT*, and *FLORAL4* (Causse *et al.*, 2002, 2023; Tieman *et al.*, 2010; Capel *et al.*, 2015; Tikunov *et al.*, 2020; Martina *et al.*, 2021). Another key component of consumer satisfaction is the texture of fruit which is massively affected by ripening status. The mutation responsible for uniform fruit ripening, now present in all modern cultivars, was identified by Powell *et al.* (2012). This mutation is encoded by a Golden 2-like transcription factor gene that regulates chloroplast development in fruits. Its reduced photosynthetic capacity has been linked to lower fruit sugar content, potentially contributing to the inferior taste of modern varieties. Since the discovery of long-shelf-life genes (Vrebalov *et al.*, 2002), only a few genes influencing fruit firmness have been identified. Notably, a mutation in pectate lyase was found to correspond to a QTL associated with fruit firmness (Uluisik *et al.*, 2016; Wang *et al.*, 2019).

It is of course not just taste but the nutritive value of our foods that is important. Plants supply not only macronutrients such as carbohydrates, fats, and proteins, but also essential micronutrients such as vitamins (A, B, C, some D, E, and K), most essential minerals, and fiber (Martin and Li, 2017). The human body can store vitamins to varying degrees, with vitamins A, D, and B_12_ being stored in substantial amounts. As a consequence, an adult’s diet may lack vitamins A and D for several months and in some cases, such as B_12_, for years, before a deficiency condition develops. Vitamin D helps prevent deficiency-related diseases that impact skeletal development and is converted into compounds with steroid hormone activity, playing a role in signaling across various organs. Li *et al.* (2022) engineered tomatoes to accumulate provitamin D_3_ through genome editing, targeting 7-dehydrocholesterol reductase within the duplicate pathway for cholesterol/SGA biosynthesis which channels carbon towards the production of vitamin D. It is yet to be seen if natural variation in the levels of vitamin D exists in tomato; however, evaluation of the same populations as described above has identified a broad number of QTLs for vitamins C and E as well as for lycopene and other carotenoids, with clear candidate genes underlying most of these (Liu *et al.*, 2003; Schauer *et al.*, 2006; Fraser *et al.*, 2007; Stevens *et al.*, 2007; Almeida *et al.*, 2011). Similarly, Quadrana *et al*. (2014) discovered that the gene responsible for a key tomato vitamin E trait, VTE3(1), is regulated by DNA methylation in its promoter region, where a SINE retrotransposon influences the activity of the gene, resulting in natural variations that affect vitamin E levels (Quadrana *et al.*, 2014).

Steroidal glycoalkaloids (SGAs) are a class of terpenoids—the most diverse group of plant secondary metabolites. SGAs are widely distributed across various plant species, particularly within the *Solanum* genus. While recent studies have uncovered several key genes (known as GAME genes) involved in SGA biosynthesis, much research is needed to fully elucidate the complete biosynthetic pathway of these compounds (Itkin *et al.*, 2011). Notably, an SGA mQTL was localized to a chromosomal region containing 14 genes, including a known SGA gene cluster. Szymański *et al.* (2020) performed an integrative mQTL and eQTL analysis in tomato fruit using 580 lines from combined BIL and IL populations. The analysis revealed a key enzymatic step involving GAME5, a UDP-glycosyltransferase that catalyzes the conversion of acetoxy-hydroxy-tomatine into esculeoside A. Additionally, Kazachkova *et al.* (2021) identified nine bitter-tasting tomato varieties using 150 resequenced genomes and genotyping a 650-tomato core collection. These bitter varieties lack a key gene, *GORKY*, which encodes a transporter responsible for moving α-tomatine from the vacuole to the cytosol—a necessary step for its conversion into non-bitter forms.

**Fig. 1.**
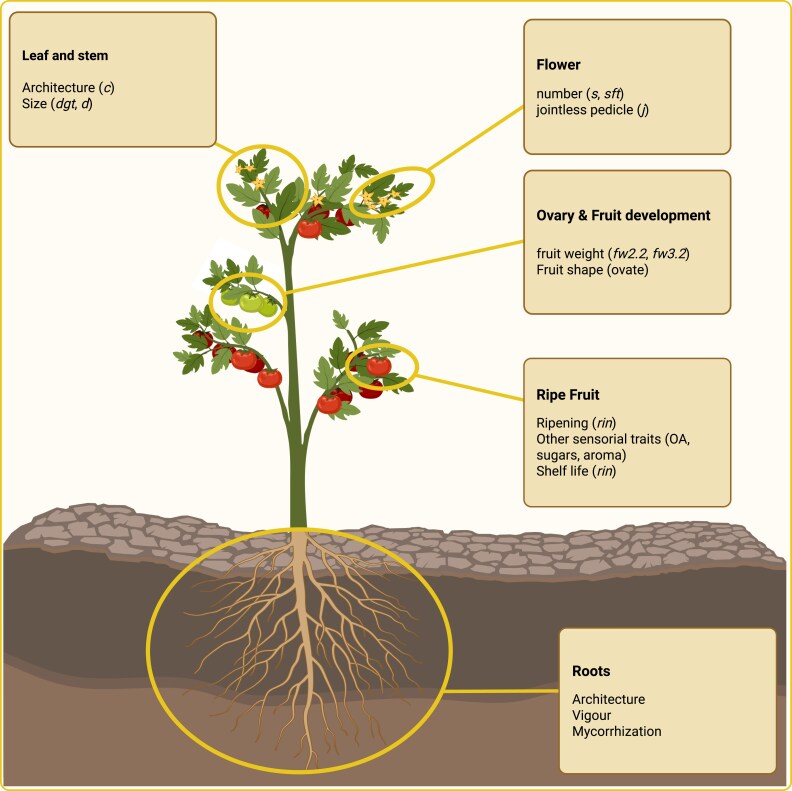
Visualization of some of the QTLs identified across various tomato tissues and phenotypic traits studied for their agricultural significance. Created in BioRender. Karakas, E. (2025) https://BioRender.com/ixvon0t.

In summary, since the pioneering work of Steve Tanskley and colleagues, who developed the first high-density molecular marker map (Tanksley *et al.*, 1992), numerous genes responsible for these mutations have been mapped and positionally cloned (Causse and Grandillo, 2016). These genes influence traits such as plant architecture [e.g. the *self-pruning* (*sp*) mutation (Pnueli *et al.*, 1998), fruit color (*del*; Ronen *et al.*, 1999); *Green ripe* (*Gr*; Barry *et al.*, 2005); and *white flower* (*wf*; Galpaz *et al.*, 2006)], fruit ripening and shelf-life [*ripening inhibitor* (*rin*; Vrebalov *et al.*, 2002) and Gr (Barry *et al.*, 2005)], and abscission [*jointless* (*j*; Mao *et al.* (2000)] (Fig. 1). Since the first cloning of the Pto gene responsible for resistance to *Pseudomonas* in tomatoes, >30 disease resistance genes have been mapped, and the majority of these have been cloned using positional cloning techniques (Foolad and Panthee, 2012).

Alongside major mutations, several QTLs influencing fruit size, shape, color intensity, firmness, and composition have been mapped. With advances in high-throughput metabolomics, the levels of hundreds of primary and secondary metabolites have been analyzed (Schauer *et al.*, 2005b, 2008; Carrari *et al.*, 2006; Bauchet *et al.*, 2014; Alseekh *et al.*, 2015, 2017, 2020; Tieman *et al.*, 2017). For tomato sensory quality, numerous QTLs related to sensory traits and volatile organic compounds (VOCs) have also been mapped (Causse *et al.*, 2002; Tieman *et al.*, 2007), with a few successfully cloned through positional cloning.

## Metabolic genome-wide association study

Tomatoes are cultivated worldwide in a wide range of diverse conditions, leading to a vast array of traits of interest for this species. Previous genome resequencing and pan-genome studies in tomato have captured both intra- and inter-species diversity across wild and cultivated varieties, shedding light on the domestication history of the tomato. These studies have been instrumental in identifying candidate genes associated with desirable traits, such as enhanced fruit flavor (Liu *et al.*, 2002; Tieman *et al.*, 2017; Gao *et al.*, 2019). Beyond fruit size and yield, breeding efforts have targeted fruit shape and composition, disease resistance, adaptation to new growing conditions, and tolerance to abiotic stress.

Following the release of the tomato reference genome, genome-wide association study (GWAS) analysis has been rapidly adopted to investigate a range of traits, including fruit composition and plant architecture. GWAS involves genotyping a genetically diverse group of individuals, taking into account linkage disequilibrium (LD)—the extent to which an allele of one SNP is correlated with or co-inherited alongside an allele of another SNP within a population. The first study panel consisted of accessions from cultivated tomato, cherry tomato, and the wild species *S. pimpinellifolium* (Sauvage *et al.*, 2014). Collections of unrelated accessions often show structural patterns that may hinder the detection of significant associations or result in false positives, reducing the reliability of GWAS for identifying true associations.

**Fig. 2.**
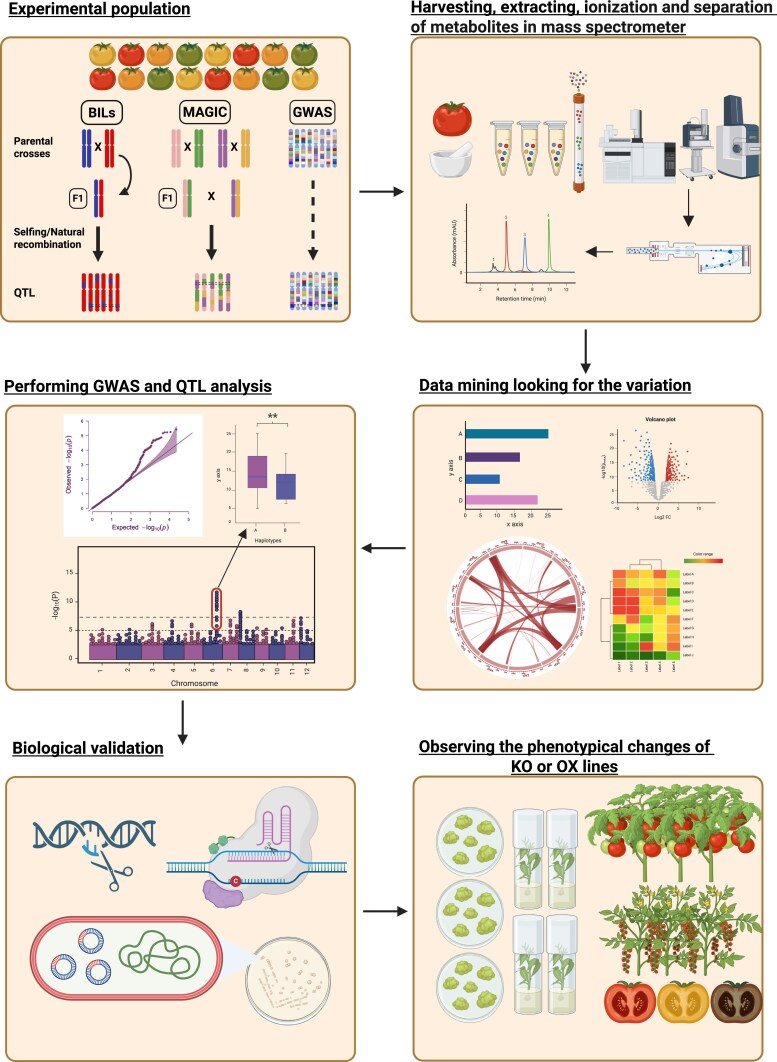
Flowchart outlining the sample processing pipeline. Starting with the experimental population, sample harvesting and extraction for MS, data analysis for the variation, genome-wide association study (GWAS), and quantitative trait locus (QTL) mapping for genetic associations, biological validation for confirming the findings, and the last step is the observation of the phenotypical changes of knockout (KO) or overexpression (OX) lines. Created in BioRender. Karakas, E. (2025) https://BioRender.com/ufeuy48.

The mapping populations used for tomato gene discovery are diverse, showcasing distinct yet complementary traits. The genomic era, with more affordable high-throughput sequencing capabilities, has made it easier to access numerous polymorphisms between individuals, even among closely related relatives. This progress has driven the development of new mapping population designs in tomatoes, with dense genetic maps that improve the accuracy and effectiveness of QTL detection. In addition to the traditional bi-parental populations and IL sets used for gene and QTL discovery, new segregating populations have recently been developed in tomatoes, including BILs and multi-parent advanced generation intercross (MAGIC) populations (Ofner *et al.*, 2016; Brog *et al.*, 2019; Burgos *et al.*, 2021) (Fig. 2). A limitation of using bi-parental mapping populations is that the small number of alleles tested may not accurately represent the natural variation within the crop (Fernie *et al.*, 2006; Ranc *et al.*, 2008; Blanca *et al.*, 2015). To address this, complementary approaches utilizing multi-parental mapping populations and panels assembled for GWAS aim to capture a broader proportion of existing genetic diversity.

The potential of GWAS has been illustrated in tomatoes, where multiple studies have unraveled the genetic basis of traits associated with fruit size and quality (Xu *et al.*, 2011; Ranc *et al.*, 2012; Bauchet *et al.*, 2014; Sauvage *et al.*, 2014; Sacco *et al.*, 2015). These studies leveraged an expanding number of markers—ranging from hundreds to several thousand—enhancing the detection of associated markers. In parallel, the linear models used in these analyses were progressively refined to account for confounding factors such as kinship and population structure, thereby reducing false-positive associations. To further improve candidate gene identification, Sauvage *et al.* (2014) applied a multi-locus mixed model, as described by Segura *et al.* (2012). However, these studies rely on publicly available genetic resources that differ genetically from the elite germplasm used to breed new tomato varieties, making it challenging to directly apply their findings to breeding programs.

A GWAS conducted on 96 tomato lines including Italian and Latin American landraces, as well as wild and modern varieties, revealed 20 significant associations linked to seven fruit traits: β-carotene, FW, *trans*-lycopene, titratable acidity, ascorbic acid, phenolic compounds, and pH (Ruggieri *et al.*, 2014). Sauvage *et al.* (2014) analyzed 163 diverse tomato lines, including *S. lycopersicum*, *S. lycopersicum* var. *cerasiforme*, and *S. pimpinellifolium*, identifying 44 significant loci associated with 19 traits, such as volatiles, sugars, and acids. This study demonstrated that the genetic control of metabolic traits varies widely, with some traits being influenced by a few genes. such as two loci explaining 74.3% of the variation in fruit dehydroascorbate levels. while others involve many genes, as seen with five loci accounting for 33.2% of the variation in ascorbate levels. Another GWAS was conducted on 123 cherry tomato lines and 51 large-fruited lines, which were evaluated for 28 volatile compounds, and 125 significant associations were identified. However, the study relied on only 182 simple sequence repeat (SSR) markers, which limited its statistical power (Zhang *et al.*, 2015). Zhao *et al.* (2016) performed a GWAS on 174 tomato lines, including 123 *S. lycopersicum* accessions and 51 *S. lycopersicum* var. *cerasiforme* accessions, identifying 58 significant associations related to sugars and organic acids. Tieman *et al.* (2017) conducted a GWAS on 398 tomato lines, encompassing modern, heirloom, and wild varieties, and identified 251 significant associations across 20 traits, including sugars, acids, and 15 VOCs. Their findings revealed that modern tomato varieties contain significantly lower levels of key flavor compounds compared with heirloom varieties.

As alluded to in the above section, the introgression of a QTL identified in these ILs has enabled plant breeders to enhance soluble solids (Brix) levels in commercial tomato varieties, significantly increasing tomato yield (Fridman *et al.*, 2004). A recent phenotype-guided screen of >7900 global tomato accessions identified novel loci for complex traits such as fruit weight and Brix, demonstrating that phenotype-guided germplasm pre-selection can uncover valuable genetic targets for breeding high-Brix, high-yield tomatoes (Zemach *et al.*, 2023). This is likely to be due to the enrichment of rare alleles that this strategy brings. However, it is important to note that the trade-off between yield and sugar content was also broken via recurrent selection (Yamamoto *et al.*, 2016) and in cases where allelic variation of a kinase that phosphorylates the enzyme sucrose synthesis were identified (Zhang *et al.*, 2024; Fernie and Martinez-Rivas, 2025).

A system-biology analysis incorporating genomic, transcriptomic, and metabolomic data from 610 tomato lines—encompassing 42 lines from wild species and 568 lines from the red-fruited clade (*S. pimpinellifolium*, *S. lycopersicum* var. *cerasiforme*, and *S. lycopersicum*)— revealed that breeding for producer-specific traits such as fruit size influences the metabolome (Zhu *et al.*, 2018). This study revealed that while fruit size genes themselves may not directly alter traits such as primary metabolite content, linked genes might contribute to these metabolic changes. This highlights the need for precision molecular breeding strategies to minimize the effects of linkage drag. Another GWAS analysis involving 192 tomato lines identified 41 significant loci associated with six fruit traits: fruit shape, fruit color, pericarp thickness, fruit weight, fruit height, and fruit width (Phan *et al.*, 2019). Similarly, a study utilizing GWAS and sweep analyses examined 166 tomato accessions to investigate traits affected by selection during domestication and the transition to *S. lycopersicum* var. *lycopersicum* (SLL) from *S. lycopersicum* var. *cerasiforme* (SLC) and *S. pimpinellifolium* (SP) (Razifard *et al.*, 2020). The authors found that reduced soluble solids were strongly associated with selection during these transitions. The analyzed population included SP from its South American region of origin, SLC from South America and Mesoamerica, and SLL landraces from Mesoamerica. Additionally, the researchers discovered that loci related to fruit size (locule number) and citric acid levels overlapped with selective sweeps during both northward expansion events of SLC, suggesting that these phenotypic changes were propably driven by selection rather than genetic drift (Razifard *et al.*, 2020). Another study examined genetic variations in five previously cloned tomato flavor genes (*LIN5*, *ALMT9*, *AAT1*, *CXE1*, and *LOXC*) using a collection of 166 accessions from South and Central America (Pereira *et al.*, 2021). This study revealed significant genetic diversity at these loci, including novel haplotypes absent in cultivated germplasm. By investigating functional causative polymorphisms and utilizing long-read genome assemblies, the researchers resolved a gene duplication at the *LOXC* locus that influences the accumulation of lipid-derived volatiles. These findings align with earlier reports of flavor-enhancing haplotypes being lost during the domestication and cultivation of tomatoes. A summary of the major genes and QTLs identified across these studies, along with the traits they influence and associated references, is presented in Table 1.

**Table 1.** The summary of major genes and QTLs associated with tomato traits

References	Trait/focus	Genes/QTLs identified and findings	Population/material used	Method or/instrument used
Eshed and Zamir (1995)	Fruit weight	Three linked QTLs	ILs from *S. pennellii×S. lycopersicum*	Fine mapping
Chakrabarti *et al.* (2013)	Fruit weight	*fw3.2* (cytochrome P450)	*S. lycopersicum*	Fine mapping and cloning
Mu *et al*. (2017)	Fruit weight	*fw11.2* (cell size regulator)	NILs	Gene functional validation
Tieman *et al*. (2006)	Aroma volatiles	*LeAADC*	Cultivated tomato varieties	Metabolite profiling; GC-MS
Tikunov *et al*. (2013)	Aroma	*NSGT1*	Cultivated tomato varieties	Combinatorial omics
Uluisik *et al*. (2016)	Fruit Firmness	Pectate lyase	NILs	QTL mapping, mutation analysis
Li *et al.* (2022)	Vitamin D	7-Dehydrocholesterol reductase	Genome-edited tomatoes	CRISPR, metabolic profiling; MALDI
Phan *et al.* (2019)	Fruit shape, color, weight, height, width and pericarp tickness	41 loci associated with six fruit traits	GWAS	GWAS, SNP genotyping, mixed linear model (MLM)
Razifard *et al.* (2020)	Domestication and selective sweeps	Selection linked to lower soluble solids, fruit size, and citric acid during northward expansion	GWAS	GWAS, sweep analysis
Pereira *et al.* (2021)	Fruit weight	Six fruit weight QTLs		QTL mapping
Itkin *et al.* (2011)	SGA	GAME genes	Tomato cultivars	Metabolite profiling; LC-MS, transcriptome analysis
Szymański *et al.* (2020)	SGA	GAME5	BIL and IL populations	mQTL, eQTL, and RNA-seq
Kazachkova *et al.* (2021)	Bitter taste	GORKY transporter gene	150 resequenced, 650 core tomato lines	whole-genome resequencing, genotyping, GWAS

In summary, tomatoes are cultivated worldwide under diverse conditions, leading to significant genetic variation. Advances in genome resequencing and pan-genome studies have provided insights into tomato domestication and identified key genes influencing traits such as fruit flavor, disease resistance, and stress tolerance. GWAS analyses have further expanded our understanding of tomato genetics, uncovering association between genetic markers and important traits such as fruit composition and plant architecture. Recent GWAS analyses have identified numerous loci associated with traits such as fruit size, metabolic composition, and fruit yield, demonstrating the potential for improving commercial tomato varieties. Integrating genomic, transcriptomic, and metabolomic data has also highlighted the complex interactions between breeding selection and metabolic changes. GWAS has provided valuable insights and offers new opportunities to refine trait selection and enhance tomato breeding efforts.

## Conclusions and perspective

This article discusses recent advances in high-throughput metabolomics and functional genetics for analyzing primary and secondary metabolites in tomato such as sugars, organic acids, vitamins, volatiles, and carotenoids. Studies on key ripening mutants, transcription factors, and hormone signaling pathways have deepened our understanding of regulatory networks, while research into metabolic QTLs and GWAS have revealed the genetic underpinnings of fruit composition, flavor, yield, and texture. Furthermore, the application of genome editing and fine-mapping strategies has led to the identification of genes with potential for improving fruit quality and nutritional value.

Despite these significant advancements, many aspects of tomato metabolism remain to be fully elucidated. Future research integrating multi-omics approaches with precise genetic interventions will be essential to uncover additional regulatory mechanisms. Such efforts will not only enhance our fundamental understanding of tomato ripening but also pave the way for targeted breeding strategies aimed at improving yield, shelf-life, flavor, and nutritional content. As new tools and techniques continue to evolve, the study of tomato metabolism will remain at the forefront of plant biology, offering valuable insights for both scientific exploration and agricultural innovation.
